# Is it really organic? Credibility factors of organic food–A systematic review and bibliometric analysis

**DOI:** 10.1371/journal.pone.0266855

**Published:** 2022-04-14

**Authors:** László Bendegúz Nagy, Zoltán Lakner, Ágoston Temesi

**Affiliations:** Department of Agricultural Business and Economics, Institute of Agricultural and Food Economics, Hungarian University of Agriculture and Life Sciences, Budapest, Hungary; Università degli Studi di Milano, ITALY

## Abstract

Consumer trust and organic food product credibility play a crucial role in understanding consumer behavior. The aim of this review is to identify extrinsic factors which influence consumers’ perceived trust in organic food. The research was conducted based on the PRISMA guidelines. During our search, 429 articles were found, from which 55 studies were selected for further analysis. To assess the connection between the selected articles, a bibliometric analysis was done with VOSViewer and CitNetExplorer software. The following factors were identified as influencing the credibility of organic food: labeling, certification, place of purchase, country of origin, brand, price, communication, product category, packaging. From these, labeling, certification, and country of origin are well-researched factors in relation to credibility. The significance of the other discovered factors is supported; nonetheless, further research is needed to evaluate their effect on consumer trust.

## Introduction

The importance of organic food is well indicated by the steadily growing market. As sustainability is more and more in the focus of food product development, organic food is becoming a successful concept in the food industry [1]. Whilst in 2008, the organic food market reached 50,9 billion USD [2], the sales of organic food doubled in only a decade, up to 119 billion USD in 2019 [3].

This growth in organic food sales can be attributed to an increased demand for organic food. The vast majority of this demand originates from North America and Europe, nonetheless, local organic markets are rising in Asia, Latin America, and Africa [1]. On account of the increasing demand for organic food, consumer trust has gained great interest among researchers [4]. However, no review article has been written on this particular topic so far.

Credibility is a relatively new research field in the context of consumable products. Green et al. [5], Plasek & Temesi [6] and Küster-Boluda & Vila [7] examined credibility in the case of alternative medicine, functional food, and low-fat food, respectively. Other researchers have explored fields related to food products in terms of credibility. Anders et al. [8] examined it within third-party certification in the food supply chain, Kumar & Polonsky [9] researched it from food retailer perspective.

Organic food can be defined based on Kahl et al.’s [10] definition: “Organic food is produced within a regulated and certified production process.” According to them, food can be described by intrinsic or extrinsic quality attributes. These attributes are strongly related to consumer expectations and trust [11].

Organic food is considered as a credence good, because there is an information asymmetry between the consumers and producers [12]. In the case of credence quality, the consumer of a product can not fully evaluate the quality of a particular good [13]. In terms of organic food, it means that the presence or absence of the organic attributes is not detectable by consumers even after purchase and consumption of the product [12].

The most widely accepted definition of trust comes from Rousseau et al. [14]: “a psychological state comprising the intention to accept vulnerability based upon positive expectations of the intention or behaviour of another.” From our viewpoint, it means that the consumers’ tolerance for ambiguity is increased as a result of an inner assurance or conviction [15]. According to Thorsøe et al. [15] there is a strong link and dependence between trust and credibility, because actors, such as producers or retailers, must be credible to generate trust in consumers, although they can not control the consumers’ perception, which can generate distrust.

## Research methodology

Our purpose in this review is to find all extrinsic, product-related factors which determine credibility and trust in organic food products. To detect those factors, we used PRISMA guidelines for this review. PRISMA enables review authors to summarize evidence in a selected field accurately and reliably [16]. There is no existing review protocol for this kind of research field.

**Fig 1:**
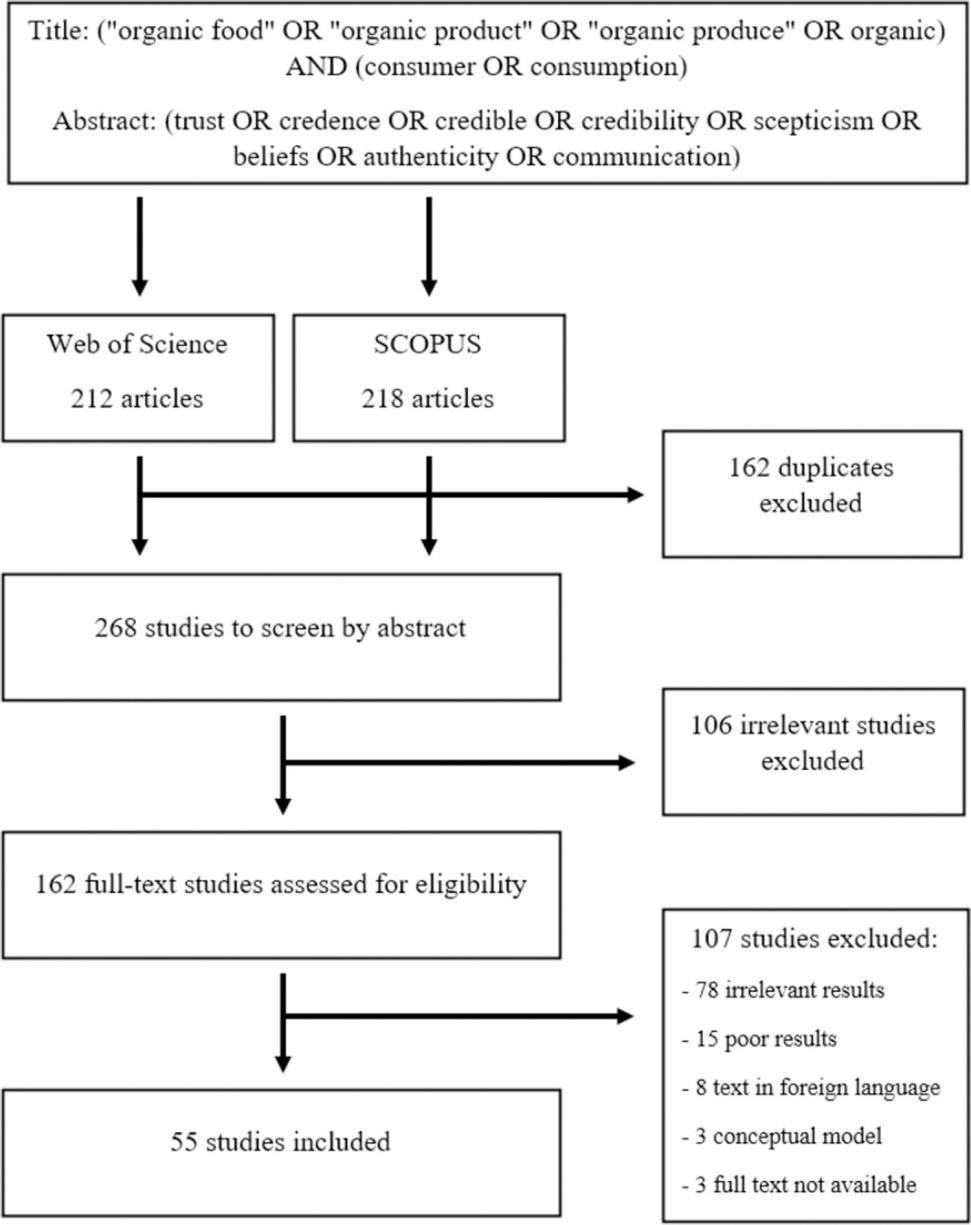
Search words and search method.

For this review, we used Web of Science and SCOPUS search engines, as those databases considered the widest and recommended sources in our research field [17]. We conducted the searches during October 2021, the last search was done on 15^th^ October 2021. To find all relevant articles about the credibility factors of organic food, we used several search phrases. The composition of search expressions had been supported by term frequency–inverse document frequency method (TF-IDF) on some randomly chosen text from the relevant field. The term “organic food” or “organic product” or “organic produce” or “organic” had to be in the title of the article, as well as “consumer” or “consumption”. These phrases narrowed down the scope of the articles mostly to consumer-related topics of organic food. In addition, the abstracts of the articles had to contain at least one of the following phrases: “trust”, “credence”, “credible”, “credibility”, “scepticism”, “beliefs”, “authenticity” or “communication”. With the above mentioned search phrases we ran pre-tests on the Web of Science search engine which proved to be accurate to describe our research topic. We did not limit the publication date of the studies, because the earliest study that we found on this particular topic was from 2002. For these search phrases, we found 212 results in Web of Science and 218 results in SCOPUS. From these, 162 records were duplicates, which were discarded (see Fig 1).

To screen and select the articles for our review, we used Covidence online software, which enabled us to evaluate articles by two authors independently in 2 steps. In the first step, we evaluated the remaining 268 articles by reading the abstract only. In this step, we excluded 106 studies, which were irrelevant to our topic. In some cases, it was not unequivocal from the abstract if an article was relevant, so these studies were selected for the full-text assessment.

In the second step, 162 articles were assessed for eligibility by reading the full-text. During this step, 107 studies were excluded for various reasons. The most common reason was being irrelevant for our research. These articles contained the required search words, although organic food consumption behavior was not assessed in the context of credibility or trust. 15 studies were excluded because of poor results, 8 articles were in a foreign language, 3 studies included a conceptual model with no results explained and 3 articles were not accessible.

Besides the systematic review, a bibliometric analysis was conducted on the selected articles to reveal the connection between the identified credibility factors. For this purpose, two different software packages were used. VOSviewer (version 1.6.15) software is capable of visualizing networks and forming clusters, which enables further analysis [18]. CitNetExplorer (version 1.0.0) can be used to study the development of a research field, which can support the literature review [19].

## Results

Only a few research has tried to tease out all possible credibility factors. Danner & Menapace [20] found 5 authenticity-related themes: organic label, origin, retail outlet/brand, packaging, product category. Tangnatthanakrit et al. [21] proposed 5 factors, which influence organic food trust: control, competence, characteristics, communication and community. Some studies list other factors as well, like natural taste, merchandising, knowledge, scarcity, and tourism [22], although there is no evidence behind these factors as to their influence on the credibility of organic food.

From the selected, manually analyzed 55 articles (see Table 1), we identified the following 9 exogenous factors which can influence the credibility of a food product: labeling, certification, place of purchase, country of origin, brand, price, communication, product category, and packaging.

**Table 1:** Selected articles and major findings.

Source	Year	Country	Method	Sample size	Sample characteristics	Major findings
**European countries**						
Krystallis & Chryssohoidis [26]	2005	Greece	Survey	164	73.8% female; biased towards younger ages and higher educational levels	Consumers who do not trust organic labels, certifiers, and retailers are not willing to pay more for organic food
Padel & Foster [23]	2005	United Kingdom	Focus group	96	Over half were female; third in full-time employment; high proportion of academic education	Organic and not organic buyers have no trust in supermarkets in case of organic food, labels, and certification increase trust, but consumers are afraid of imported organic food
Pivato et al. [11]	2008	Italy	Structural equation modeling, survey	400	Not available	CSR activities of retailers positively influence trust in organic food
Perrini et al. [62]	2010	Italy	Survey	183	Average age was 48 years; 67.8% female; frequent shoppers	Consumers are more likely to trust private-label organic products if they consider the retailer as socially responsible
Janssen & Hamm [38]	2012a	Czech Republic, Denmark, Germany, Italy, Switzerland, United Kingdom	Choice experiment	2441	Level of education was generally high; mean household size was above average	Organic logos create consumer trust, well known and trusted logos are perceived as stricter standard and control system
Janssen & Hamm [40]	2012b	Czech Republic, Denmark, Germany, Italy, United Kingdom	Focus group, survey	149, 2042	Females and younger ages are overrepresentated	Trust in the EU organic logo and the certification behind was not very high
Gerrard et al. [39]	2013	United Kingdom	Focus group, survey	29, 410	70% females; 52% under 45 years old	Consumers trust products which have a national (Soil Association) organic logo more than the EU logo (or without a logo)
Müller & Gaus [74]	2015	Germany	Survey	145	University students	Negative media harms organic food trust
Vittersø & Tangeland [53]	2015	Norway	Survey	1987	Representative samples	Norwegian consumers trusted labeling less in 2013 than in 2000
Zander et al. [41]	2015	Estonia, France, Germany, Italy, Poland, United Kingdom	Survey	3000	Representative samples	Pragmatic organic consumers trust organic certification regardless of the country of origin, committed consumers have lower trust in global certifications
Bryła [22]	2016	Poland	Survey	1000	Representative samples	The following factors influence organic food authenticity: natural taste, product quality, label, quality sign, retailer, merchandising, appearance, knowledge, packaging, brand name, region, scarcity, and tourism
Thorsøe et al. [15]	2016	Denmark	Focus group, survey	5, 5467	Females, older ages and higher education and higher incomes are overrepresented	Danish consumers have high trust in the labeling and the certification
Perić et al. [73]	2017	Serbia, Croatia	Survey	520	Females are overrepresented	63% of Serbian and 50% of Croatian respondents do not believe advertising on organic food
Činjarevic et al. [51]	2018	Croatia	Survey	184	Females and higher education are overrepresented	Most consumers are skeptical about product claims of organic food on the labeling and advertising
Meyerding & Merz [50]	2018	Germany	Eye tracking, conjoint analysis	73	Younger ages and higher education are overrepresented	The occurrence of organic label creates trust in the product
Pedersen et al. [68]	2018	Germany	Focus group, survey	38, 255	Regular organic buyers; 68% female	The trust in the exporting country influences the organic food trust
Steffen & Doppler [60]	2018	Germany	Case study, interview	10	Older ages are overrepresented	Brand and retailer are important to a customer, although they do not believe in certificates
Vega-Zamora et al. [71]	2019	Spain	Survey	800	Not available	Communication helps to build trust towards organic food
Ladwein & Romero [61]	2021	France	Survey	316	Not representative; very diverse	Trust in retailers and producers has a positive impact on purchase intention and the authenticity of organic food
**European and non-European countries**						
Thøgersen et al. [64]	2019	Germany, France, Denmark, China, Thailand	Survey	6059	Representative sample	Country of origin is a more important quality cue than organic labeling, consumers prefer products from developed countries
Danner & Menapace [20]	2020	USA, Germany	Online comment analysis	1069	Not applicable	The authors found 5 authenticity-related themes: organic label, origin, retail outlet/brand, packaging, product category
**Non-European countries**						
Lockie et al. [75]	2002	Australia	Focus group	130	Not available	Certification is important, but processed food makes people suspicious whether it is organic
Essoussi & Zahaf [56]	2008	Canada	Focus group	6 focus groups	Younger ages are overrepresented	Labeling, certifiers are creating trust amongst consumers, they are skeptical about imported organic food, and they do not trust superstores
Essoussi & Zahaf [42]	2009	Canada	In-depth interview	21	Younger ages are overrepresented	Distribution, certification, country of origin, and labeling are related to consumers’ trust in organic food
Zepeda & Deal [55]	2009	USA	Semi-structured interview	25	Not available	Consumers do not trust organic food from Wallmart
Van Loo et al. [44]	2011	USA	Choice experiment	976	Females and higher education are overrepresented	USDA organic logo creates more trust than a generic organic logo
Chen & Lobo [48]	2012	China	Structural equation modeling, survey	960	Younger ages are overrepresented	Labeling is the most important factor influencing consumer beliefs
Sangkumchaliang & Huang [30]	2012	Thailand	Survey	390	Higher education are overrepresented	The knowledge of certification body is important to the customer to trust organic product
Tung et al. [52]	2012	Taiwan	Survey	913	Not available	Taiwanese consumers do not trust organic labels
Bruschi et al. [32]	2015	Russia	Focus group, survey	26, 160	Higher education are overrepresented	Russian consumers trust European certifications more than local ones
Hemmerling et al. [70]	2015	-	Review	277 articles	Not applicable	Packaging of certain organic food seems to be not environmentally friendly to consumers
Teng & Wang [46]	2015	Taiwan	Survey	693	Higher education are overrepresented	Labeling is significant to the creation of consumer trust
Yip & Janssen [65]	2015	China	Survey	245	Females, older ages and higher incomes are overrepresented	Hong Kong consumers found Chinese organic product less trustworthy than local and imported organic product
Bonn et al. [57]	2016	USA	Survey	471	Females and higher education are overrepresented	Consumers are more likely to purchase organic wine from a retailer they trust
Yin et al. [45]	2016	China	Survey	876	Not available	Well-known brands are trusted more than lesser-known brands, low price reduces consumer trust and certification has no significant impact on trust
Nuttavuthisit & Thøgersen [29]	2017	Thailand	Focus group, in-depth interview, survey	16, 10, 177	Higher education and income are overrepresented	General trust in the certification system in Thailand is low, consumers rely on package appearance, and the retail store
Yue et al. [63]	2017	China	Laboratory experiment	120	Younger ages are overrepresented	Media richness of website and review lengths of product impacts the trust in organic food in case of E-commerce
Kim et al. [43]	2018	USA	Consumer panel analysis	154308	Representative sample	USDA organic labeling is more credible than third party organic certification
Konuk [59]	2018	Turkey	Survey	352	Age group 31–40 are overrepresented	Store image influences the trust in private-label organic food
Sobhanifard [49]	2018	Iran	Survey	546	Median age was 38 years; 58% females	Product claims, psychological security, and doubt are the main components of organic food trust
Chen et al. [35]	2019	China	Survey	576	55% females	Chinese consumers trust organic products with organic labels from developed countries
Hwang & Chung [58]	2019	USA	Survey	318	68% females; median age was 49 years	Consumers’ perception of retailer’s store quality positively influences organic food fit
Lee et al. [47]	2019	Taiwan	Survey	928	66% females; most representation was from 41–50 years old	Labeling, local production, and price premium affects the trust in organic food
Yadav et al. [33]	2019	India	In-depth interview	34	Males are overrepresented	There are many different organic certifiers in India, which confuse consumers, and there are no known brands of organic food that they can trust
Yin et al. [66]	2019	China	Choice experiment	853	Income level was slightly higher than the average	Trust in organic food depends on the country of origin and certifiers
Kantamaturapoj & Marshall [72]	2020	Thailand	In-depth interview	9	Not available	Certification and retail communication is key to consumer trust
Lian & Rajadurai [36]	2020	Malaysia	Survey	390	54% females; most representation was from 40–49 years old	Malaysian consumers trust their national organic logo, myOrganic
Liang & Lim [54]	2020	Taiwan	Survey	592	Females and higher education are overrepresented	Nutritional values on the labeling enhance trust in the organic labels
Watanabe et al. [31]	2020	Brazil	Survey	382	Undergraduate students are overrepresented	Brazilians have a lack of trust in institutions and companies, which influences consumer trust
Yormirzoev et al. [67]	2020	Russia	Survey	608	58% females; median age was 36 years	Consumer trust organic milk from the EU more than from Russia
Truong et al. [34]	2021	Vietnam	Interview	27	93% female; median age was 35 years	Vietnamese consumers are sceptic in local certifications’ authenticity, USDA certificate create more trust. Bigger retailers are seen more trustworthy in case of organic vegetables.
Tangnatthanakrit et al. [21]	2021	Thailand	Survey	319	Females between age of 30 and 49	Authors proposed 5 factors, which influence organic food trust: control, competence, characteristics, communication and community. Community had the biggest impact on trust, control, competence and communication does not influence trust
Watanabe et al. [76]	2021	Brazil	Survey	349	80% females; 42.7% aged from 18 to 25 years	Consumers’ trust varies on fresh produce category and certification. They trust in organic vegetables better than fruit.
Yang et al. [37]	2021	China	Choice experiment	450	Males and younger ages are overrepresented	Contrary to other food products, in case of oolong tea Chinese consumers prefer Chinese organic certification
Yu et al. [69]	2021	China	Survey	269	Females and higher education are overrepresented	CSR activities of organic food companies can positively influence consumer trust of organic food

### Bibliometric analysis

**Fig 2:**
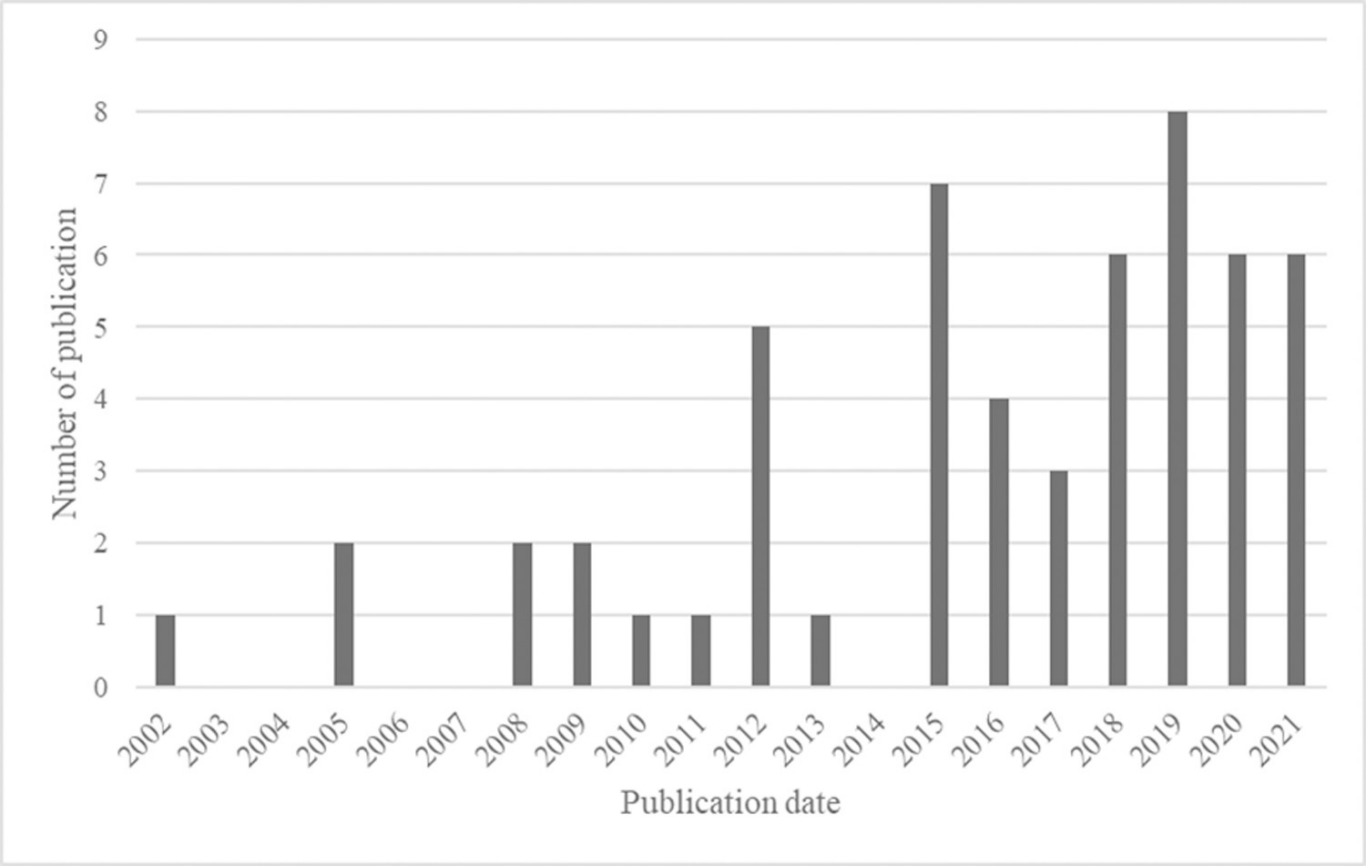
Distribution of articles by publication date.

Of the selected 55 papers, more than half were published after 2016, which indicates the current interest in this research field (see Fig 2). Only 7 studies were conducted before 2010.

In terms of location, most of the research was conducted in European countries. More than 1/3 of the articles report results from Asian countries, and only 8 papers write about North American consumers, which does not represent the actual size of the organic food market of these continents. There are 2 articles from Brazil and Australia each, which provide valuable results as well.

**Fig 3:**
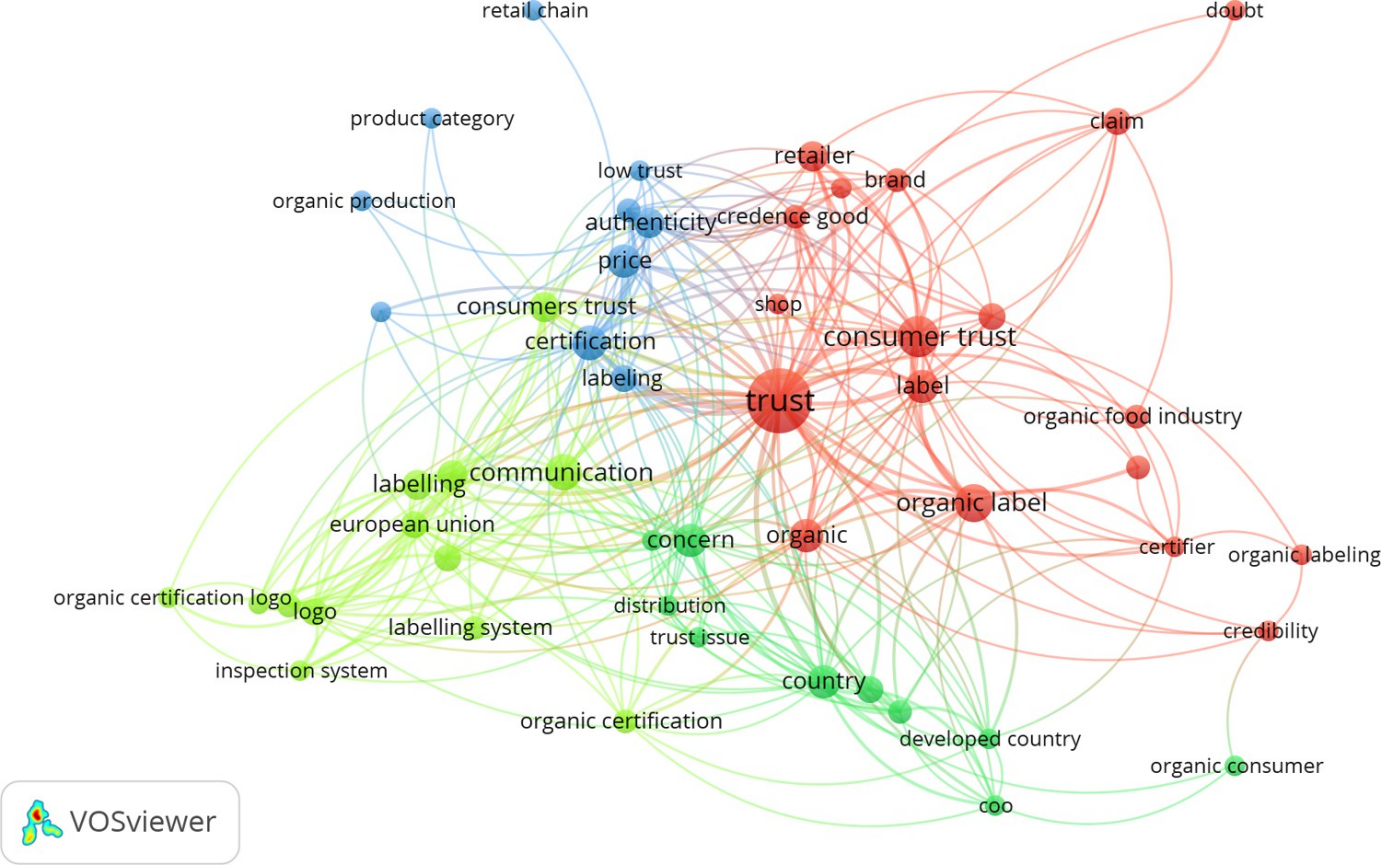
Network visualization of credibility factors.

Fig 3 shows the connections and co-occurrence of the identified credibility factors. With the VOSviewer software, the terms related to credibility, trust, and the influencing factors were chosen from the abstracts. The size of each circle represents the number of occurrences in the selected articles, and co-occurrence is illustrated by the distance between the circles.

Based on the connections of the 9 identified credibility factors, 4 clusters could be identified. The red cluster contains the most terms, and trust is the most relevant term in the selected papers. Trust is strongly related to organic label and shop, although retailer and brand are also significant to trust, which correlates with the findings of Padel & Foster [23]. In the blue cluster, labeling, certification, price, authenticity, and low trust are very closely related to each other. Retail chain and product category also belong to this cluster, which supports the results of Danner & Menapace [20].

Communication, which is mentioned by Tangnatthanakrit et al. [21], is in the middle of the light green cluster, and it is very close to labeling and concern, although concern belongs to the green cluster. Logo, inspection, and certification also appear in the light green cluster with the European Union, which shows that most of the research related to organic logos was about the EU organic logo. Concern, distribution, trust issue, and country are the main terms in the green cluster. These terms represent the connection between country of origin and consumer concerns. Although these clusters do not represent each credibility factor, this analysis is a good indicator of the connections between the factors.

**Fig 4:**
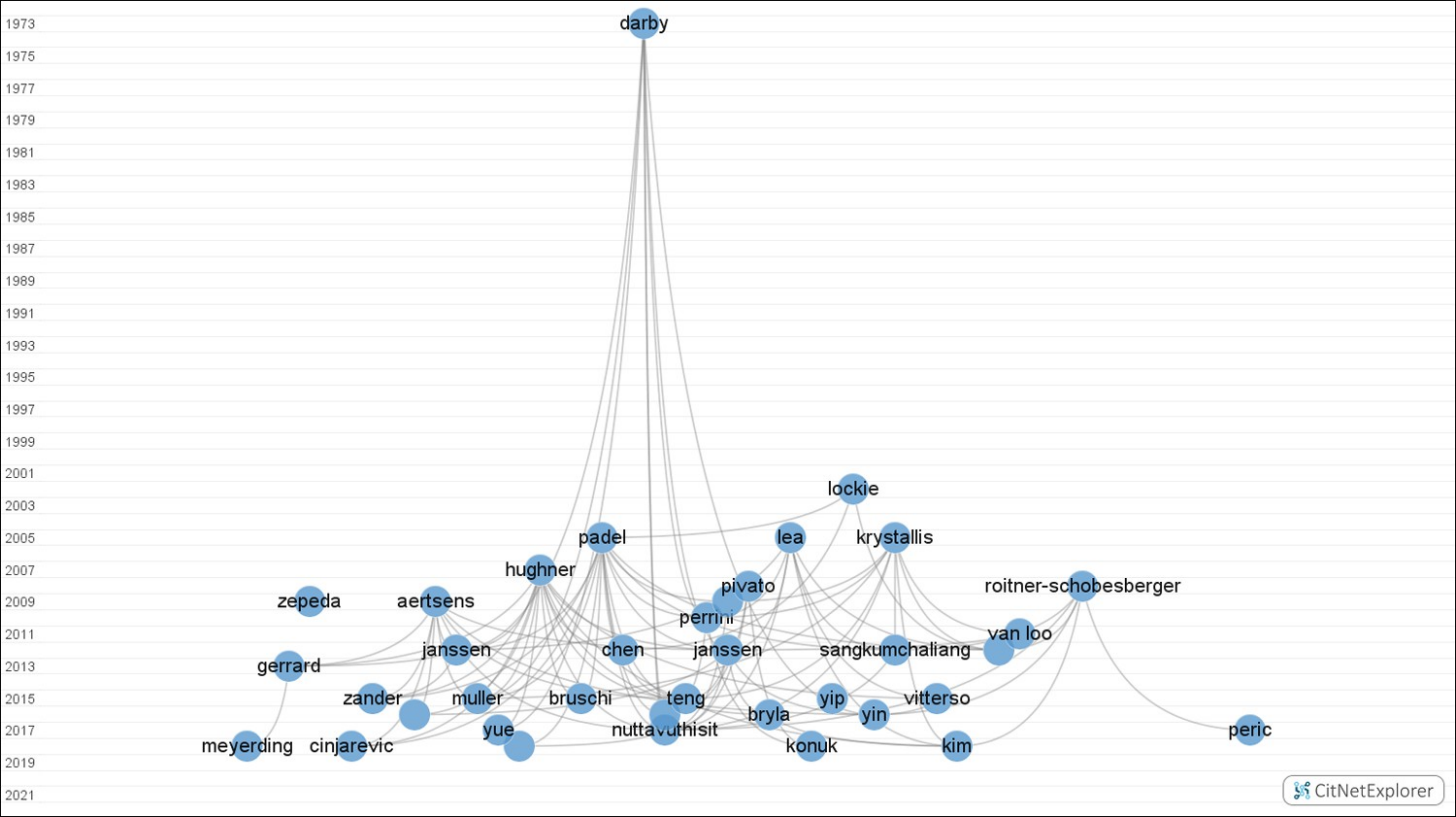
Network visualization of citations.

The visualization capability of CitNetExplorer has been a useful tool because it allowed us to find the most relevant publications and investigate the intellectual roots of our research topic. With the CitNetExplorer, connections between the citations of the chosen 55 papers can be visualized, as seen in Fig 4.

Each circle represents a publication, and publications are labeled with the first author’s last name. Vertical location shows publication year, with old articles at the top and new publications at the bottom. In the horizontal direction, publications are arranged according to citation relationships. Highly cited publications that take into account direct and indirect citation relationships tend to be closer to each other horizontally. Publications that are less relevant with respect to other citations are further away [24].

Nine publications were cited 10 or more times, from which 3 papers are included in the review. The article by Padel & Foster [23] was cited most frequently, namely 21 times. They investigated qualitatively consumers purchasing decisions of organic food. From our perspective, their most important findings were that labeling, certification and the country of origin play an important role in the perceived trust of organic food, which tend to be the major factors in later publications as well.

Almost the same amount, 20 papers cited the review of Hughner et al. [25], in which they explore the reasons why people buy organic food. This publication does not mention trust related factors of organic food, although it gives important conclusions about the nature of organic food consumption.

Four articles were cited 13 times, from which 3 were published before 2010. Krystallis & Chryssohoidis [26] discussed the importance of labeling, certification and the place of purchase from the credibility perspective. Lea & Worsley [27] investigated Australian consumers’ beliefs about organic food. Aertsens et al.’s [28] review is discussing the personal determinants of organic food consumption.

Nuttavuthisit & Thøgersen’s [29] article was published in 2017, although it was cited 13 times, which shows the relevance of this paper to our topic. As they did a qualitative research about the consumer trust in Thailand, it offers important statements about the credibility factors of organic food in emerging countries.

The oldest cited publication is from 1973, written by Darby & Karni [13]. In their publication, they clarify the meaning of the credence attribute, which explains the high citation number.

### Certification

Half of the selected articles—28 by number—mention certification as one of the most important factors influencing the credibility of organic food. Organic logos are discussed in this part because these logos represent the certification itself, and usually, it is a legal requirement as well.

Evaluating the selected research, it can be observed that generally, consumers have lower trust towards organic food with a certification from a developing country. For example, general trust in the certification system is low in Thailand [27], but it can create trust if consumers know about the certification body [30]. The preference for certification from a developed country and lack of trust in the local certifiers can be seen in the case of Brazilian [31], Russian [32], Indian [33], Vietnamese [34], and Chinese [35] consumers.

We observed some opposite results as well. Malaysian consumers trust their national organic logo, myOrganic [36]. In the case of oolong tea, Chinese consumers prefer Chinese organic certification [37].

In the case of European consumers, we can see a more nuanced picture. Janssen & Hamm [38] examined consumer reactions to organic logos in six European countries. Their results show that organic logos create consumer trust; well known and trusted logos are perceived by the consumers as having stricter standards and control system behind them. Consumers from the United Kingdom trust their national logo more than the European Union organic logo or an organic product without any logo [39]. Czech, Danish, German, Italian and UK consumers also have lower trust towards European Union organic logo compared to their national organic logo [40], although it is important to mention, that compulsory EU logo usage was recently implemented by the time of data collection of the research. Based on the research of Zander et al. [41], which was performed in six European countries, trust in the certification system and organic logo can be differentiated by types of consumers. Regular and occasional organic consumers trust organic certification regardless of its origin, on the other hand, consumers who have higher knowledge and involvement towards organic food have lower trust in global certifications.

The organic food market is different in the United States and Canada, although consumer attitudes are similar to the European market. Certification plays an important role in the credibility of organic food in the case of Canadian consumers [42]. Both Kim et al. [43] and Van Loo et al. [44] agree that in the case of consumers from the United States, an USDA organic logo creates more trust than any generic organic logo.

Overall, most of the research shows that certification has a significant role in the credibility of organic food, but Yin et al. [45] question the importance of it. According to them, certification has no impact on consumer trust in the case of milk products. Tangnatthanakrit et al. [21] obtained similar results during their research in Thailand.

### Labeling

Labeling is as important for a product to be credible as certification. Labeling is a general term in this case since it partly covers other factors as well, like certification, brand, or packaging. There is no clear distinction amongst the authors between labeling and organic logos; some research considers organic logos as part of the labeling. In this review, we consider labeling as information about the product displayed on the packaging, and organic logos were discussed separately in the previous sub-section.

According to Teng & Wang [46], Essoussi & Zahaf [42], Lee et al. [47], Chen & Lobo [48], Padel & Foster [23], and Sobhanifard [49] labeling is significant to the creation of consumer trust in the case of organic food. Most research shows a positive relationship between labeling and credibility, although a lot of them challenge it as well. For example, Thorsøe et al. [15] proved that Danish consumers trusted organic labeling, Meyerding & Merz [50] used an eye-tracking method and found evidence that the presence of an organic label created trust in the product. On the other hand, based on Činjarević et al. [51], Croatian consumers are skeptical about the organic claims on labeling; Tung et al. [52] agree that Taiwanese consumers do not trust organic labels.

Trust in labeling can change over time, as Vittersø & Tangeland’s [53] study in Norway shows. They compared data from 2000 and 2013, and found that Norwegian consumers had more trust in organic labeling in 2000 than in 2013. Also, the content of the labeling is not indifferent for credibility. Nutritional values on the labeling enhance trust in the organic labels, based on the research of Liang & Lim [54].

### Place of purchase

Of the selected articles, nineteen pay attention to the place of purchase as a factor influencing credibility. The majority of those papers, namely 16 cover only retailers, 2 paper mention supermarkets, and only 1 inspects trust from the perspective of online shops. Unfortunately, we did not find any research on organic specialty shops, direct sale, or farmers’ market, although these sales channels can be important in the case of organic food.

We found miscellaneous results regarding supermarkets and organic food trust. Mostly in the United States, United Kingdom, and Canada, consumers have low trust in organic food if it is sold in a superstore [23, 55, 56]. Nonetheless, research has confirmed that positive consumer perception of a retailer has a positive impact on the credibility of the organic food sold there [57–61]. In their work, Pivato et al. [11] show a positive relationship between the corporate social responsibility (CSR) activities of a retailer and the trust in the organic food sold in their stores.

Many retailers are selling organic food under private labels, so there is a bit of an overlap between the place of purchase and the branding of a product. According to Perrini et al. [62] consumers are more likely to trust private-label organic products if they consider the retailer as socially responsible.

Organic food retail could not avoid the spread of e-commerce, although research is very limited in this field. Yue et al. [63] investigated the influence of online product presentation on organic chicken breast. Based on their research, the media richness of online product presentation and review lengths of organic products impact the trust in organic food.

### Country of origin

The origin of organic food has significant importance for perceived credibility. This topic was partly discussed in subsection Certification, because organic food is usually certified in the country where it comes from. As in the case of certification, we can see differences between consumers of developed and developing countries, although based on Thøgersen et al. [64] country of origin is an even more important cue for consumers than organic labeling both in developed and developing countries.

According to Lee et al. [47], Yip & Janssen [65], and Thorsøe et al. [15] Taiwanese, Hong Kong, and Danish consumers have higher trust in local organic food compared to imported ones. Canadian and UK consumers are skeptical about imported organic food [23, 56].

Based on the findings of Bruschi et al. [32], Chen et al. [35], Yin et al. [66] and Yormirzoev et al. [67], the opposite reaction can be seen by consumers from developing countries. Chinese consumers trust organic food from developed countries [35, 66], Russian consumers trust European organic food [32, 67]. These findings can be explained with the research of Pedersen et al. [68]. Based on their results, the image and trust in the exporting country affect the trust in the organic food they export.

### Other factors

Brand, price, communication, and product category were also identified as influencing factors of credibility, although only a few articles discuss these factors.

Brand is a trust-building factor in the case of organic food. Yin et al. [45] found that well-known brands are trusted more compared to lesser-known brands. According to Steffen & Doppler [60], the branding of organic food creates more trust than certification. CSR activities of organic food companies can positively influence consumer trust of organic food [69]. The lack of known brands can cause trust issues in certain markets [33].

The effect of price on organic food authenticity is supported by the bibliometric analysis. Research has proved that the high price of organic food is a barrier to consumption [70]. On the other hand, Lee et al. [47] point out that premium price affects trust in organic food, and Yin et al. [45] proved that in the case of organic milk, low price reduced consumer trust in the product. This is true the other way around: consumers are not willing to pay more for organic food if they do not trust it [26].

Product-level and retail-level communication help to build trust toward organic food [71, 72], although Perić et al. [73] disagree with it. According to them, 63% of Serbian and 50% of Croatian consumers do not believe advertisements on organic food, which derives from the general mistrust in the media and advertising. Müller & Gaus [74] investigated the effect of media on organic food trust. Based on their research, negative media harms the credibility of organic food products.

The credibility of certain organic product categories is questionable for consumers. According to Lockie et al. [75], processed organic food makes consumers suspicious whether it is in fact organic. Consumers’ trust can varies on fresh produce category. Based on Watanabe et al. [76], consumers trust organic vegetables better than organic fruit.

Packaging seems to influence consumers’ trust in organic food, although there is very limited research on this topic. Danner & Menapace [20] identified packaging as an influencing factor, although its impact on credibility was questioned only by the consumers of the German-speaking countries, whereas USA consumers did not find it a credibility issue. German, Austrian and Swiss consumers believe that in the case of organic fruit and vegetable, plastic packaging makes them appear ‘less organic’ [20]. In their review, Hemmerling et al. [70] confirm the theory that packaging seems to be not environmentally friendly in the eye of consumers, as it is against the idea of organic food, although packaging can also be useful because it can indicate the organic status of the product. Nuttavuthisit & Thøgersen [29] mention that consumers rely on the appearance of the packaging when they assess the credibility of organic food.

## Conclusions and future perspectives

The goal of our research was to identify the factors which influence the perceived credibility of organic food products. In the review, we could find 9 different product-related factors, not equally well-researched, and there are blind spots where further research is needed.

The interest in organic food is growing, however we can see a shift from developed to developing countries in terms of geographical focus of the articles. This shift and geographical difference in consumer attitudes could be detected by almost all identified factors of organic food credibility.

Certification is one of the most important factor to build consumer trust, as certification covers all those activities where compliance with organic requirements are assessed, so that should be a guarantee for consumers. Existing research shows a clear pattern regarding the credibility of certification bodies in different countries. Certifications from developed countries are much more trusted compared to certifications from developing countries.

Labeling has the role to inform consumers about the product. Without this information, consumers can not be sure if a product is organic. Besides certification, labeling is crucial to inform consumers about the organic characteristics of a product, which transfers the credence attribute to a search attribute. The importance of labeling can be explained with the fact, that labels contain most of the information about the product, so consumers can assess the product from other perspectives (eg. nutritional values, origin, ingredients, etc), which might influence perceived trust.

Labeling is well researched factor, however there are some kind of loose products, where the lack of labeling is common practice, like fruit and vegetables or bakery products. In those cases, credibility might be questioned by consumers, so research on these products is desirable.

The results of the credibility aspects of the country of origin seem to correlate with the results on certification, and the findings are strongly related to the results of the bibliometric analysis. Organic products from developing countries can cause doubt in consumers both from developed and developing countries, which might indicate the general low institutional trust in these countries.

Research on the effect of place of purchase proves its importance, although it is incomplete in several areas. According to Ökobarometer [77], German consumers mostly buy organic food in supermarkets and discounters, although traditional markets, specialty shops, and direct purchase also play an important role in organic food retail. However, these sales channels were not taken into account in the existing research, thus further research is needed.

In the case of certification, labeling, and country of origin, the findings of existing research seem to provide enough evidence to draw a reliable conclusion. All of these factors play an important role in the perception of trust towards organic food.

Brand was less-researched in relation to credibility, but all evidence shows that it has a positive impact on the authenticity of organic food. Similarly, not much research has investigated the effect of price, communication, product category, and packaging of organic food on credibility, therefore further research is needed in connection to these factors. There are certain product attributes, which were not evaluated by previous papers, but the authors assumed that they might have a strong effect on organic food trust. As food packaging is getting in the scope of sustainability, it would be interesting to compare the influence of different type of packaging on the level of trust. Also, color of the package can influence consumers’ perceptions of organic food.

The main aim of this review was to cover all the credibility factors of organic food; however, there are many limitations of this work. Identification of the credibility factors was based on the selected papers, therefore there might be other factors influencing credibility in the case of organic food and other articles, which cover the topic of this review. The reviewed articles are covering a wide range of research methods and geographical locations, so the samples are not homogenous.

## Supporting information

S1 File PRISMA checklist. (DOC)

S2 File List of reviewed articles. (XLSX)

S3 File Dataset for bibliometric analysis. (TXT)
